# Predicting kidney failure risk without albuminuria: implications in chronic kidney disease

**DOI:** 10.1136/bmjmed-2026-002559

**Published:** 2026-02-02

**Authors:** Heather Y Walker, Jennifer S Lees

**Affiliations:** 1 Population Health and Genomics, University of Dundee, Dundee, UK; 2 School of Cardiovascular and Metabolic Health, University of Glasgow, Glasgow, UK; 3 Glasgow Renal and Transplant Unit, NHS Greater Glasgow and Clyde, Glasgow, UK

**Keywords:** Kidney failure, chronic, Risk management, Preventive medicine

A new kidney failure risk equation that does not require albuminuria testing has the potential to improve health equity and access, but albuminuria testing is important beyond prediction of kidney failure, write **Walker** and **Lees**

With novel treatment strategies available to delay or prevent kidney failure, early identification of individuals at the highest risk is a key priority to improve health outcomes and reduce healthcare costs.^1^ The kidney failure risk equation, calculated using age, sex, estimated glomerular filtration rate, and urine albumin to creatinine ratio (uACR), is the most extensively validated and widely used kidney failure prognostic model in patients with chronic kidney disease^2 3^; however, suboptimal uACR testing limits its implementation.^4 5^ In a large observational cohort study published in *BMJ Medicine* (doi:10.1136/bmjmed-2025-001950), Cleary and colleagues developed a risk prediction model for kidney failure at five years, in individuals with chronic kidney disease that uses routinely collected data and does not require uACR testing.^6^

Using data from more than 116 000 adults in the Stockholm Creatinine Measurements (SCREAM) project, the authors identified that 77% of individuals with chronic kidney disease stages 3-4 (estimated glomerular filtration rate 15-60 mL/min/1.73 m^2^) did not have a uACR result within 12 months before or three months after diagnosis of chronic kidney disease to allow calculation of the kidney failure risk equation. These findings—showing inadequate uACR testing among people with or at high risk of chronic kidney disease—are consistent with explorations in the UK and internationally.^4 7^ Cleary and colleagues developed and validated a 10 variable model that does not require uACR, incorporating estimated glomerular filtration rate, age, diabetes, sex, atrial fibrillation, anti-hypertensive drugs, peripheral artery disease, decline in estimated glomerular filtration rate, acute kidney injury, and hypertension.

This new model demonstrated high discrimination, good calibration, and consistent predictive performance across the study population and in subgroups by age, sex, diabetes status, disease stage, and uACR availability. Comparatively, the kidney failure risk equation had marginally better discrimination and clinical usefulness than the new model, but showed considerable miscalibration and could only be calculated for 23% of the cohort. These findings suggest that the kidney failure risk equation should be preferentially used, but where uACR is missing, the new model performs better than the kidney failure risk equation with three variables that excludes uACR.

This new model provides a tool that could be automated within primary care to identify and risk stratify individuals at high risk of kidney failure, who require risk factor modification, therapeutic optimisation, or specialist nephrology input. Acknowledging lower rates of uACR testing in subgroups of chronic kidney disease (including female individuals, older adults, and those with a higher estimated glomerular filtration rate),^4^ the applicability of this new model to all individuals with disease stage three or four has the potential to improve health equity and access. Nevertheless, its implementation would introduce yet another risk prediction tool to primary care, where awareness of the kidney failure risk equation anecdotally remains limited and implementation variable despite inclusion in current UK national^8^ and international^9^ guidance.

Albuminuria testing is prognostically important beyond prediction of kidney failure: higher uACR is strongly associated with cardiovascular disease, mortality, and other adverse outcomes.^10^ Furthermore, albuminuria is used as a qualifying criterion novel drug treatments for chronic kidney disease, including sodium glucose cotransporter 2 inhibitors and non-steroidal mineralocorticoid antagonists. Research and healthcare policy must consider strategies to enhance uACR testing in high risk populations, including but not limited to patients with diabetes, hypertension, and cardiovascular disease.^1^ The new model in this study highlights the potential use of angiotensin converting enzyme inhibitors and angiotensin 2 receptor blocker prescriptions as a proxy for albuminuria. Automated flagging of high risk individuals when prescribing these drug treatments could be used to prompt timely uACR testing.

Future research requires external validation of this new model across diverse populations, with periodic revalidation to account for evolving healthcare practices and to ensure sustained accuracy and applicability, as highlighted by the researchers. Observed trends in kidney failure underestimation in patients with uACR data—and overestimation in those without such data—suggest that additional unmeasured factors influence both clinical decisions to perform uACR testing and kidney failure risk, which warrant additional exploration. Furthermore, this study demonstrated variability in estimated glomerular filtration rate follow-up intervals, highlighting the need for studies exploring models to predict monitoring requirements and standardise practices.

In conclusion, this study underscores the potential of a kidney failure risk prediction model to improve prognostic assessment in chronic kidney disease, particularly when uACR testing is not immediately available. While the kidney failure risk equation remains the optimal and simplest tool when complete data are available, the proposed model offers a pragmatic alternative for broader implementation in primary care including automation within electronic health record systems. Future work should focus on external validation and strategies to enhance uACR testing, ensuring that risk prediction complements comprehensive management of chronic kidney disorder.
